# Infiltration of biomineral templates for nanostructured polypyrrole†

**DOI:** 10.1039/c8ra07805j

**Published:** 2018-10-02

**Authors:** A. Göppert, H. Cölfen

**Affiliations:** Department of Chemistry, Physical Chemistry, University of Konstanz Universitätsstrasse 10, Box 714 D-78457 Konstanz Germany helmut.coelfen@uni-konstanz.de

## Abstract

Biomineral templates like sea urchin spine, nacre or eggshell were applied in the polymerisation of pyrrole. The insufficient infiltration of pyrrole into the CaCO_3_ structure of the biomineral templates was improved using three different and universally applicable approaches and the electrochemical properties of the received polypyrrole were examined.

## Abstract 2

The insufficient infiltration of pyrrole into the CaCO_3_ structure of biomineral templates was improved using three universally applicable approaches.

## Graphical abstract

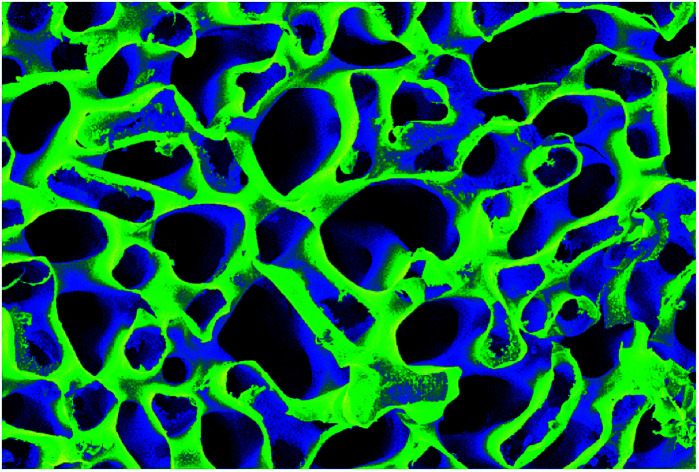

## Introduction

**Fig. 1:**
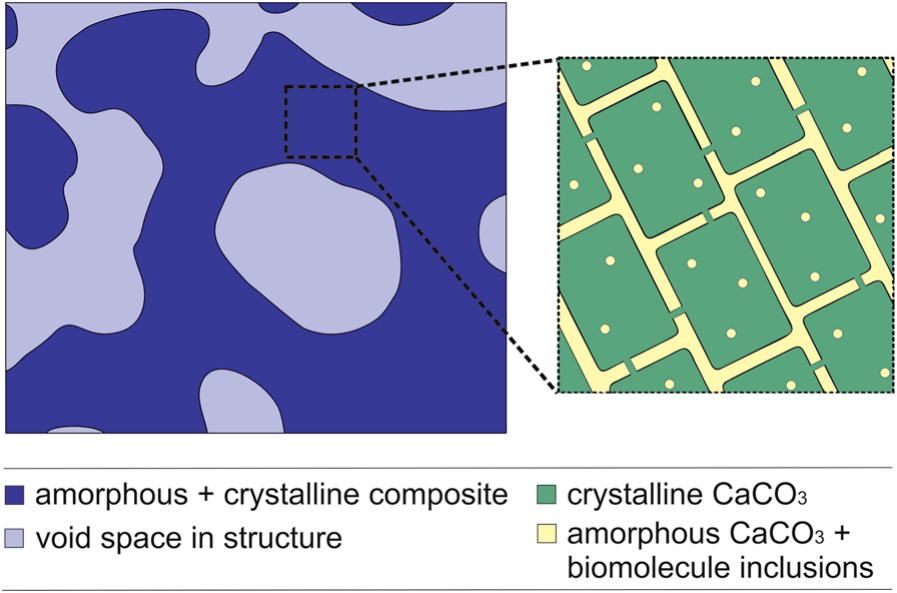
The schematic structure of a sea urchin spine according to Seto *et al.*^1^. (left) The structure on the micrometre scale in dark blue. The light blue domains clarify the pores on the micrometre scale. (right) The CaCO_3_ nanocrystals with amorphous CaCO_3_ and organic inclusions. After the removal of the organic inclusions the yellow parts between the nanocrystals are partly available as pores on the nanometre scale.

Biominerals have developed in a variety of organisms for at least 3500 million years and have different functions *e.g.* a shell for protection against predators or the skeleton of vertebrates as a supporting structure.^2,3^ Biominerals have complex morphologies and most of them show a hierarchical structure consisting of organic and inorganic components that results in special properties, like magnetic and optical properties or an increased mechanical stability, like in nacre.^2–5^ Amongst 60 different biominerals CaCO_3_ is the most abundant.^6^ The CaCO_3_ structure of some biominerals is composed of CaCO_3_ nanocrystals with biomolecule inclusions (Fig. 1).^1,7,8^ The inspiration from the complex structure of some biominerals is an intriguing approach to develop functional and multifunctional materials with remarkable properties.^9,10^ To transfer the hierarchical structure of biominerals to new materials, it is possible to use biominerals as templates which can be realised with two different strategies. The first strategy uses the organic matrix of a biomineral as a template to mineralize a new material inside. Siglreitmeier *et al.* demineralised the CaCO_3_ structure of a shell to receive the organic matrix. Within this organic matrix they infiltrated gelatine and synthesised magnetite nanoparticles to form a new organic–inorganic hybrid material.^11^ The second strategy is to dissolve the organic matrix of a biomineral and to utilize the obtained mineral structure as a template after infiltration. Imai *et al.* demonstrated that it is possible to infiltrate molecules like dyes between the nanocrystals inside a biomineral template.^12,13^ Later, Imai *et al.* used the CaCO_3_ structure of biominerals as templates for polymerisations.^14–17^ Generally templates are used often in polymerisations, as it is not easy to control the morphology of an organic polymer since they are often poorly soluble or difficult to process.^12,15,18^ A biomineral as a template allows to transfer the hierarchical structure of biominerals, consisting of the nano- and macrostructure, to polymer materials. In general the morphology has a huge impact on the properties of a material, as in the example of biominerals.^15,19^ If the hierarchical structure with its nanostructure is transferred to a conductive polymer, it could lead to interesting electrochemical properties.^20,21^ Nanostructured conductive polymers show advantages such as a high electrical conductivity,^22,23^ a high surface area^24,25^ or a short distance for the transport of ions.^26^ The hierarchical structure could lead to a better passage of the electrolyte in a bulk material, because of its micrometre pores, which lack in a merely nanostructured material. Thus, in this work we tried to replicate the synthesis of the conductive polymer polypyrrole (PPy) with a sea urchin spine as a template as it is described elsewhere.^14,16^

## Results and discussion

**Fig. 2:**
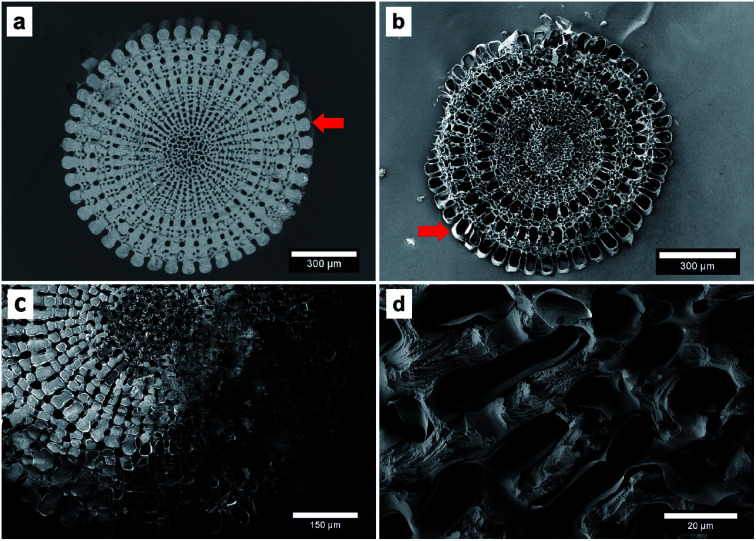
SEM images of (a) the sea urchin spine template and (b) the synthesised PPy. The PPy structure is equal to a coating of the original CaCO_3_ structure. This can be easily realised at the outer part marked with the red arrows. (c) and (d) show SEM images of the composite material consisting of CaCO_3_ from the sea urchin spine and PPy. The CaCO_3_ has been dissolved partly in this sample. (c) The light structures of CaCO_3_ can be seen. They are surrounded by a layer of PPy. The dark part of the structure is the remaining PPy where the CaCO_3_ has been dissolved already. (d) The image shows the inner part of the braces which are partly dissolved and a layer of PPy around the surface of the braces.

The process consists of four steps: preparation of the template by removing the organic components, infiltration of the pure monomer, polymerisation of the monomer and dissolving of the template. The entire process is described in detail in the ESI† and has been displayed by EDX measurements (Table 1 and S2†). The polymerisation resulted in the coating of the sea urchin spine surface with PPy on the microscale and we were not able to observe PPy inside the CaCO_3_ structure (Fig. 2). So, we assume that no polymerisation has taken place inside the CaCO_3_ structure, meaning the pores between the nanocrystals of the biomineral (Fig. 1). These results suggest that the pyrrole (Py) monomer could not be infiltrated into the nanopores of the CaCO_3_ sea urchin spine structure. We also achieved comparable results with other biominerals, such as nacre, corals and eggshells which also consist of nanostructured building blocks with incorporated organic polymer and therefore are suitable as templates, too.^7^

**Table 1:** Results of EDX measurements in at% for the sea urchin spine as template (CaCO_3_), the composite material (CaCO_3_, PPy) and the PPy after the dissolution of the template

Sample	Calcium	Carbon	Oxygen	Copper	Chlorine	Nitrogen
Template	15.34	24.23	60.43	—	—	—
Composite	19.63	22.70	54.75	0.79	0.88	1.25
PPy	—	65.03	15.66	3.33	0.33	15.53

Nevertheless it would be desirable to infiltrate the nanopores between the nanocrystals of the biominerals, to achieve a nanostructured PPy with expected better electronic properties.^22–24,26^ Therefore, the goal of this work was to improve the infiltration of the monomer Py into different biominerals to achieve a nanostructured PPy. The infiltration could be enhanced with three independent methods which can be applied to various infiltration problems.

**Fig. 3:**
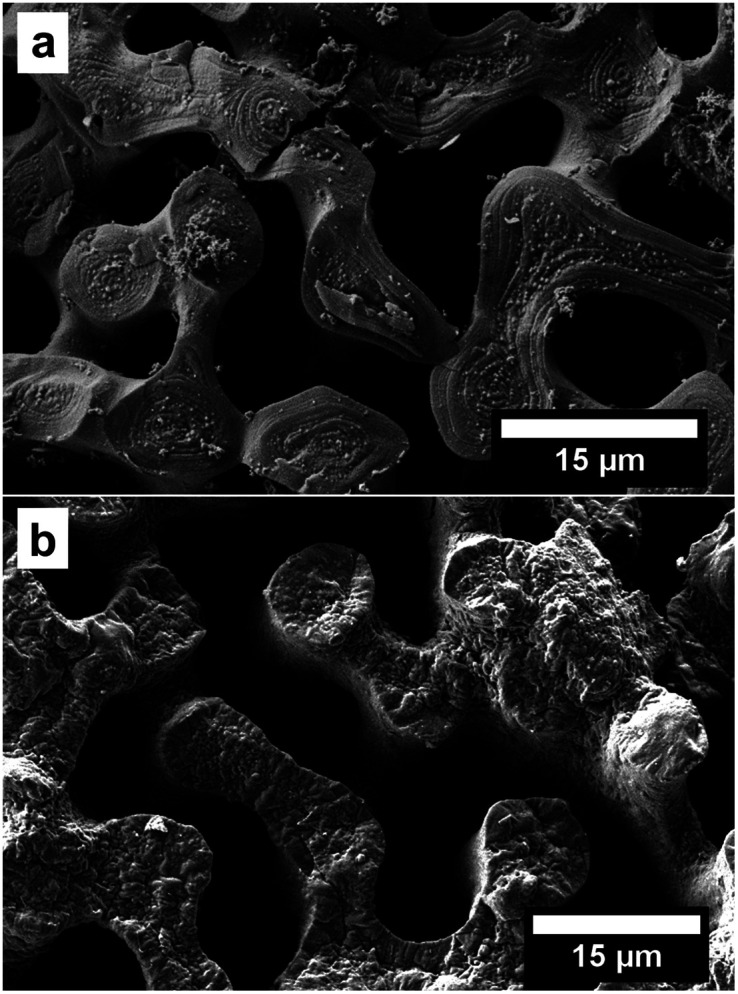
SEM images of the PPy structure (cross section) after the dissolution of the CaCO_3_ template (sea urchin spine). (a) The Py was infiltrated with an additional pressure of 1 bar as described in ESI 1.2.† (b) The Py was infiltrated from the gaseous phase after the oxidant has been infiltrated as described in ESI 1.3.†

In the first approach we used an increased pressure of 1 bar, as an external force, to improve the infiltration of the Py into the nanopores of the CaCO_3_ sea urchin spine scaffold. Porous systems can't be infiltrated spontaneously by a non-wetting liquid under atmospheric pressure, based on the capillary effect.^27^ However it is possible to infiltrate these systems under an increased pressure, which is dependent on the pore size.^28^ Using this technique, we were able to partially polymerise Py monomers inside the CaCO_3_ sea urchin spine structure (Fig. 3a). This technique is straightforward, yet an experimental set-up for the required higher pressures is needed.

**Fig. 4:**
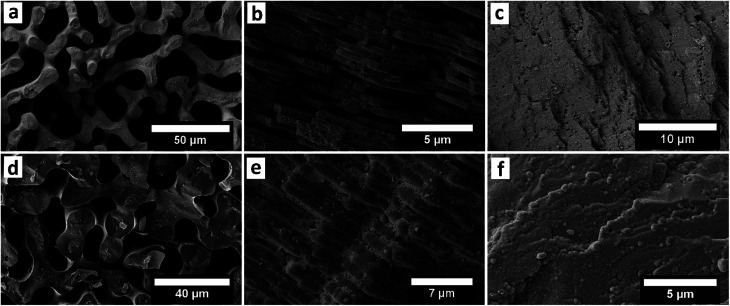
SEM images of the cross section of the CaCO_3_ templates of (a) sea urchin spine (b) nacre (c) eggshell. The pictures show the structures on the micrometre scale and the nanopores of the templates are not resolved. The second row shows the cross section of the obtained PPy structures polymerised in (d) sea urchin spine after the dissolution of the CaCO_3_ template, (e) nacre and (f) eggshell before the dissolution of the CaCO_3_ template.

**Fig. 5:**
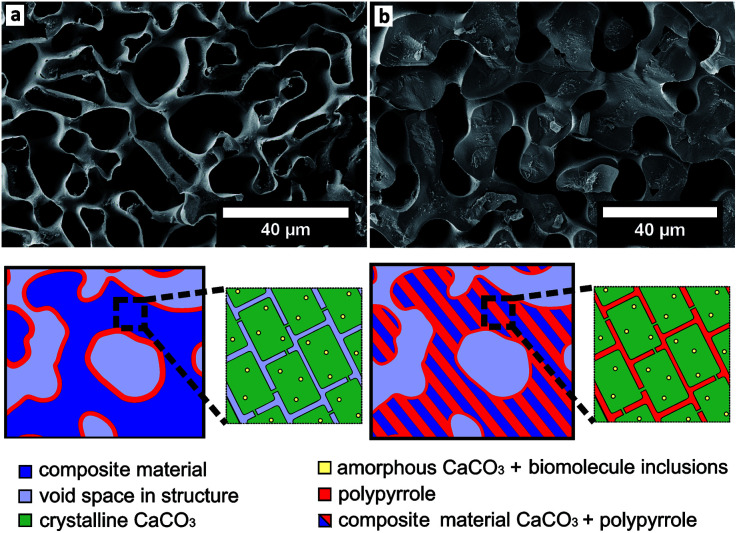
(a) SEM image of PPy structure (cross section) after the dissolution of the CaCO_3_ template (sea urchin spine). The PPy was infiltrated and polymerised as described elsewhere.^14,16^ The sketch under the picture shows the schematic structure of a sea urchin spine template. (left) The structure on the micrometre scale in dark blue. The light blue domains clarify the pores on the micrometre scale. The location of the PPy is drawn in red, its polymerisation resulted in the coating of the template structure. (right) The CaCO_3_ nanocrystals with amorphous CaCO_3_ and organic inclusions. After the removing of the organic inclusions the light blue parts between the nanocrystals are partly available as pores on the nanometre scale. With the original synthesis the Py could not be infiltrated into these nanopores. (b) As comparison on the right side the PPy structure (cross section) after the dissolution of the CaCO_3_ template (sea urchin spine). The PPy was infiltrated as a mixture with the polar solvent methanol as described in the ESI 1.2.† The sketch under the picture shows, the Py could be infiltrated into the nanopores and thus into the complete CaCO_3_ structure. After the dissolution of the CaCO_3_ template we obtain the same macrostructure made of PPy.

In the second approach we improved the infiltration ability of the Py monomer liquid by adding a polar solvent. The infiltration ability can be described by the capillary eqn (1).1
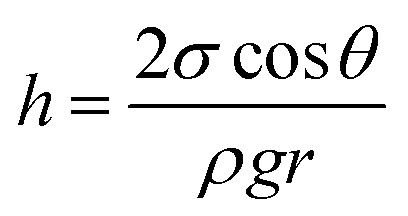
where *h* is the height of a liquid in a column, *σ* is the liquid–air surface tension, *θ* is the contact angle, *ρ* is the density of the liquid, *g* is the local acceleration due to gravity and *r* is the radius of the column. The capillary equation shows, that if we reduce the contact angle, by improving the wettability of CaCO_3_ with Py, we can increase the ability of Py to enter the nanopores. We learned from contact angle measurements that the wettability can be enhanced if the Py is mixed with a polar solvent such as methanol (Fig. S1†). After improving the wettability, we successfully infiltrated the CaCO_3_ nanostructure of the sea urchin spine with Py and polymerised inside the structure (Fig. 4a and d). The sample in Fig. 4d shows the complete sea urchin spine structure which consists solely of PPy, indicating that the polymerisation took place inside the nanopores, in contrast to the original polymerisation on the surface of the template (Fig. 5). The synthesis of PPy has been verified by ATR-IR measurements (Fig. S3 and S4†) and TGA measurements show that we were able to infiltrate 1.38 wt% of PPy into the sea urchin spine template (Fig. S5†). Afterwards we used other biominerals such as nacre, eggshell and coral to compare the results with the sea urchin spine. The infiltration of the coral unexpectedly did not work, although they are known to have a mesocrystalline structure.^29^ But the infiltration and polymerisation inside nacre and eggshell worked well with the improved wettability (Fig. 4). The handling of this approach is straight forward. Nevertheless, the dilution of the monomer might be a limiting factor in other systems. The wetting behaviour has to be sufficient for an infiltration but with a high enough concentration of the monomer. There will be one point, there the concentration of the monomer is too low for a sufficient polymerisation. In this case a possible strategy could be to use a supercritical fluid as an infiltration medium.^30,31^

The third approach is based on the capillary equation as well. We turned around the procedure and first infiltrated the oxidant CuCl_2_. Afterwards the polymerisation was started by adding the monomer Py from the gaseous phase.^32^ The contact angle measurements (Fig. S1†) showed that CaCO_3_ has a better wettability with isopropyl alcohol because it is more polar than Py. Therefore, the oxidant dissolved in isopropyl alcohol infiltrates the CaCO_3_ structure better than the pure monomer Py. An EDX measurement shows the infiltration of the CuCl_2_ into the CaCO_3_ sea urchin spine structure (Fig. S6†). The infiltration of the CuCl_2_ is more demanding, compared to the synthesis of the first and second approach. But this method showed the best outcomes regarding the reproduction of the complete sea urchin spine structure as a result of a better infiltration into the nanopores (Fig. 3b).

**Fig. 6:**
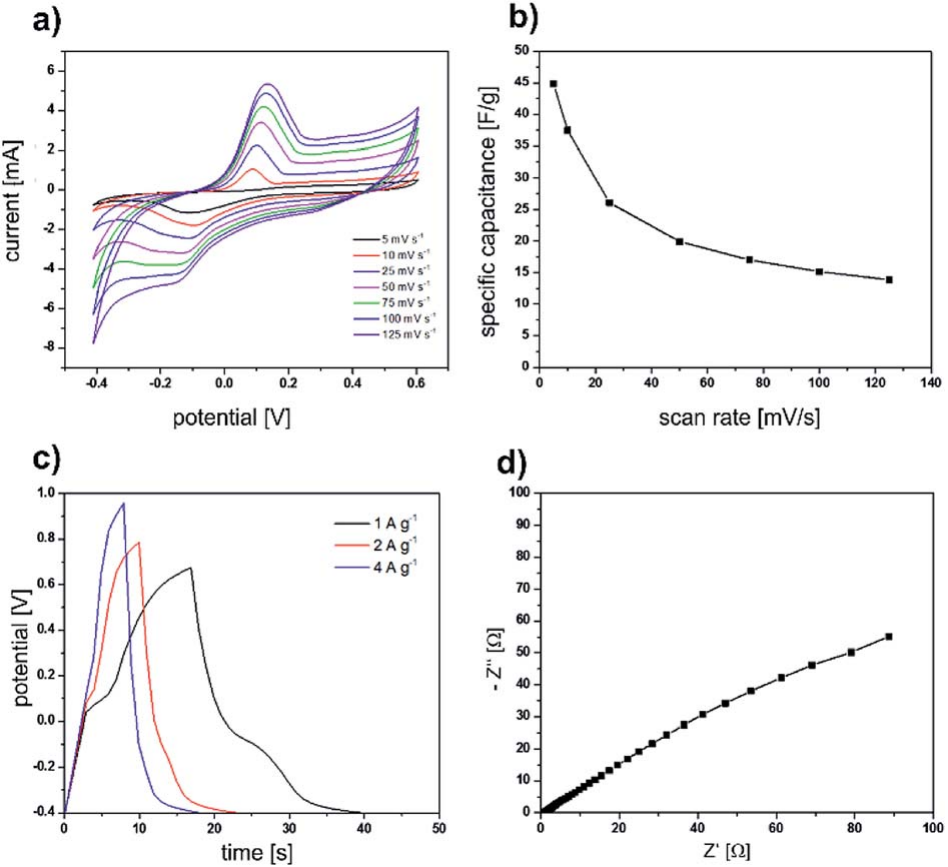
Electrochemical measurements of PPy, which was synthesised under the same conditions as the template synthesis, in solution. Results of cyclic voltammetry measurements: (a) graphs for the different scan rates (b) specific capacity calculated from the graphs *versus* the scan rates. (c) Results of galvanostatic charge/discharge curves for the different current densities. (d) Nyquist diagram. The EIS measurement was carried out over the frequency range of 0.1 Hz to 100 kHz.

**Fig. 7:**
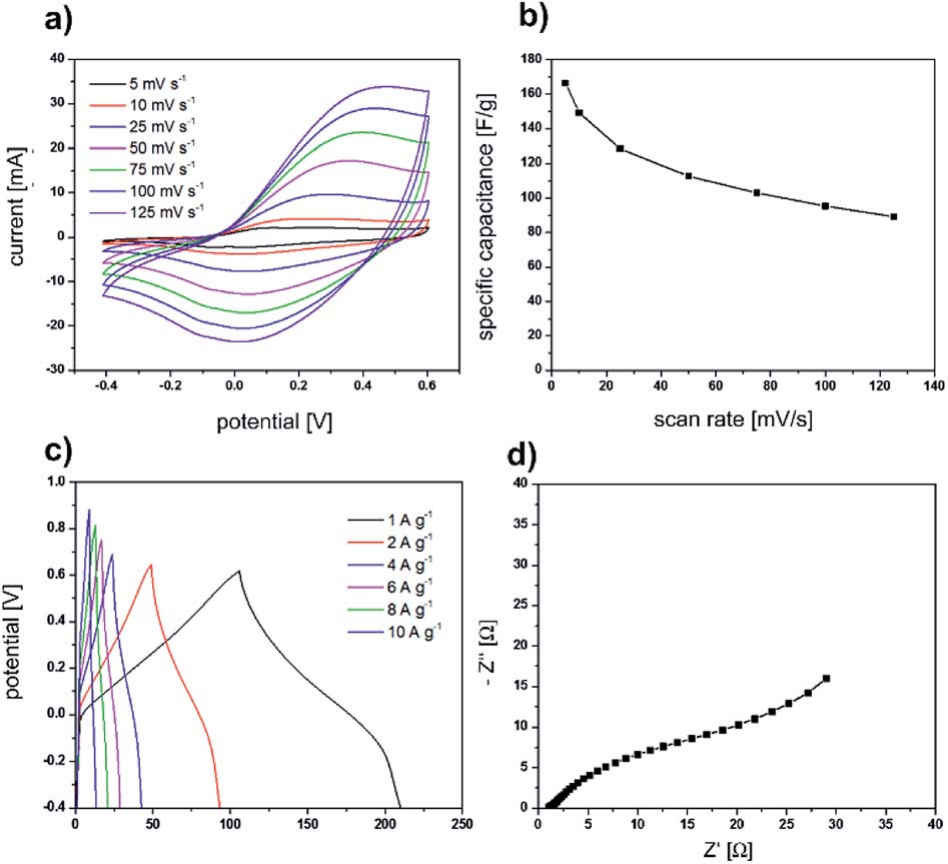
Electrochemical measurements of PPy, which was synthesised under the same conditions as the template synthesis, in solution. Results of cyclic voltammetry measurements: (a) graphs for the different scan rates (b) specific capacity calculated from the graphs *versus* the scan rates. (c) Results of galvanostatic charge/discharge curves for the different current densities. (d) Nyquist diagram. The EIS measurement was carried out over the frequency range of 0.1 Hz to 100 kHz.

After we successfully used the sea urchin spine and other biominerals as templates for the synthesis of nanostructured PPy, the electrochemical properties of the obtained PPy were analysed. The cyclic voltammetry curves (Fig. 6a and b) display a symmetric shape with a pair of redox peaks indicating the pseudocapacitive behaviour of the prepared electrodes. The obtained specific capacitance value is 45 F g^−1^ at a scan rate of 5 mV s^−1^. Furthermore, the electrochemical performance of the electrodes was evaluated using galvanostatic charge/discharge tests and EIS measurements (Fig. 6c and d). All the galvanostatic charge/discharge curves exhibit a nearly symmetric shape with a voltage plateau in the charge/discharge process which is consistent with the results obtained from the CV curves. The Nyquist curve presents a semicircular arc in the high-frequency region and a straight line in the low-frequency region. The equivalent series resistance amounts to 1.06 Ω. The large diameter of the semicircle at the high-frequency region indicates a high interfacial charge transfer resistance. The electrochemical properties are not superior compared with the results of PPy synthesised in solution under the same conditions (Fig. 7). The possible reason is, that the PPy synthesised in solution, is already nanostructured. In addition, we determined *via* BET analysis a surface area of 16.43 m^2^ g^−1^ of the PPy from the sea urchin spine (Table S1†). The surface of the PPy from solution is comparable with 13.3 m^2^ g^−1^. The BET analysis shows, that the nanostructuring of the PPy from solution is comparable to the PPy synthesised in the sea urchin spine and therefore, we could not achieve an improvement in the electrochemical properties.

## Conclusions

In summary we were able to improve the insufficient infiltration of Py into CaCO_3_ nanostructures of biomineral templates. This could be realised by three different methods as using an increased pressure during the infiltration or enhancing the wettability of the CaCO_3_ substrate with the infiltration medium. These improvements are universally applicable for other infiltration problems. With the improved infiltration of Py into CaCO_3_ nanostructures it was possible to polymerise the Py monomers inside the macro- and especially the nanopores of the biomineral templates. The hierarchical structure of the biominerals were transferred to the PPy polymer material and as PPy is a conductive polymer we analysed its electrochemical properties. Based on a working infiltration it is promising to use biominerals as templates to produce functional materials. Besides further conductive polymers, like polythiophene, a conductive replacement with gold or a replacement with wax seem to be promising.

## Conflicts of interest

There are no conflicts to declare.

## Supplementary Material

RA-008-C8RA07805J-s001
