# Supplementary material for: Infiltration of biomineral templates for nanostructured polypyrrole
Source: RSC Adv. 2018 Oct 2;8(59):33748–52. doi: 10.1039/c8ra07805j (PMC9086734; doi:10.1039/c8ra07805j)
Supplement: RA-008-C8RA07805J-s001 [file RA-008-C8RA07805J-s001.pdf]

Electronic Supplementary Material (ESI) for RSC Advances.
This journal is © The Royal Society of Chemistry 2018

Electronic Supplementary Information (ESI)

Infiltration of Biomineral Templates for Nanostructured Functional Materials

Ann-Kathrin Göppert, Helmut Cölfen*

Physical Chemistry, Department of Chemistry, University of Konstanz, Universitätsstr. 10, D-78457 Konstanz, Germany

Email: helmut.coelfen@uni-konstanz.de

Content

1. Experimental section ... 2
1.1 Preparation of biominerals as templates to remove the incorporated biological macromolecules ... 2
1.2 Infiltration of the monomer and following polymerisation ... 2
1.3 Infiltration of the oxidant and following polymerisation ... 2
1.4 Selective removal of the template ... 2
1.5 Materials ... 3
1.6 Devices ... 3
2 Characterisation ... 4
2.1 SEM and EDX ... 4
2.2 TEM ... 4
2.3 Microtome ... 4
2.4 TGA ... 4
2.5 ATR-IR ... 4
2.6 BET ... 4
2.7 Preparation of electrodes ... 4
2.8 Electrochemical measurements ... 4
3 Figures ... 5
3.1 Contact angle (Figure S1) ... 5
3.2 EDX analysis (Figure S2) ... 6
3.3 TGA (Figure S5) ... 8
3.4 EDX mapping (Figure S6) ... 9
3.5 BET surface (Table S1) ... 10

1. Experimental section

1.1 Preparation of biominerals as templates to remove the incorporated biological macromolecules

Treatment with sodium hypochlorite. The sea urchin spine, nacre and eggshell were immersed in a freshly prepared 5 wt.-% sodium hypochlorite aqueous solution. In the solution, they were treated with ultrasound for 1 h and left subsequently for 24 h at RT. The whole procedure was repeated. Afterwards the biominerals were washed with purified water and dried at RT.

The red coral was immersed in a freshly prepared 5 wt.-% sodium hypochlorite aqueous solution. In the solution, it was treated with ultrasound for 1 h and left subsequently for 3 h at RT. Afterwards the coral was washed with purified water and dried at RT.

Thermal treatment. After the treatment with sodium hypochlorite the biominerals were treated thermally. The oven was heated up from RT for 45 min, kept the temperature constant and cooled down to RT for 45 min. The temperature and treating time for the different biominerals were: sea urchin spine 450 °C 4 h, nacre 300 °C 2 h, eggshell 200 °C 2 h and red coral 200 °C for 24 h. After the procedure, the biominerals had a white colour.

1.2 Infiltration of the monomer and following polymerisation

Infiltration of the monomer: The biominerals were immersed in an excess amount of Pyrrole (Py) neat liquid for 48 h and stored at 4 °C.

Improved Infiltration with increased polarity: The biominerals were immersed in an excess amount Py mixed with a polar solvent (25 vol.-% MeOH and 75 vol.-% Py) and stored for 48 h at 4 °C.

Improved infiltration with applied pressure: The biominerals were immersed in an excess amount of Py neat liquid for 48 h and stored at 4 °C. Afterwards the biominerals, immersed in Py, were transferred to a pressure vessel. An additional pressure of 1 bar was applied for 6 h.

Polymerisation: A solution of 100 mM $CuCl_2$ in 2-propanol was prepared. The biominerals were removed from the Py liquid and the excess Py liquid on the surface of the biominerals was adsorbed by a paper towel. Before further drying, the biominerals, with infiltrated Py, were immersed in the $CuCl_2$ solution for 24 h at RT with stirring. The black colouring of the biominerals shows the successful polymerisation. After the polymerisation, the biominerals were washed with 2-propanol and dried at RT.

1.3 Infiltration of the oxidant and following polymerisation

Infiltration of $CuCl_2$: The procedure was carried out under nitrogen atmosphere. The biominerals were dried in vacuo. A solution of 100 mM $CuCl_2$ in 2-propanol was prepared and the biominerals were covered with an excess amount of this solution. After 1 h the residual solvent was evaporated. In a second step, the biominerals were covered again with the $CuCl_2$ solution and the process was repeated. Finally, the biominerals were coloured yellow.

Polymerisation in solution: The procedure was carried out under nitrogen atmosphere. The biominerals, with infiltrated $CuCl_2$, were cooled to -50 °C. A solution of 30 mg Py in 0,5 mL $CHCl_3$ was added dropwise and left there for 30 min. Subsequently the solution was removed with a syringe and remaining solvent residues were evaporated. The biominerals were allowed to warm up to RT and kept there for 24 h. The polymerisation started during the warm up, which could be recognised by the black colouring of the biominerals. Afterwards the biominerals were washed with 2-propanol and dried at RT.

Polymerisation in gaseous phase: The biominerals, with infiltrated $CuCl_2$, were transferred into a beaker and placed in the desiccator. Another beaker with Py was placed in the desiccator, too. The biomineral was treated with the Py vapour for 48 h. The polymerisation started after a few minutes, which could be recognised by the black colouring of the biominerals. Finally, the biominerals were washed with 2-popanol and dried at RT.

1.4 Selective removal of the template

After the polymerisation, the $CaCO_3$ from the biominerals was removed selectively. The materials were immersed in an aqueous 0,25 M solution of EDTA-2Na and stirred for 48 h. The pH-value of the EDTA solution has been adjusted to 10 with an aqueous 1 M NaOH solution. The procedure was repeated once. The residual polymer was cleaned by stirring it in purified water for 48 h. This procedure was repeated once. The polymer was lyophilised to preserve the polymer structure.

1.5 Materials

Biominerals: sea urchin spine (*Strongylocentrotus purpuratus*) were purchased from Marinus Scientific (Stanford, Sea Urchin Embryology); nacre (*Haliotis*) was purchased from Australian Seashells, the shells have been sandblasted to reveal the nacre; commercially available eggshell were purchased from the supermarket (*Gallus gallus domesticus*); red coral (*Corallium rubrum*) was provided by Dr. Vielzeuf from the *Centre Interdisciplinaire de Nanoscience de Marseille*

Chemicals: The chemicals were used without further purification. 2-propanol (99,5 %), chloroform (99,0 – 99,4 %), methanol (≥ 99,8 %) and pyrrole (≥ 98 %) were purchased from Sigma-Aldrich. EDTA-2Na (97 %) was purchased from Carl Roth and NaOH (1 M) from Merck.

1.6 Devices

The thermal treatment of the biominerals was carried out with a preheating oven L3/12 from Nabertherm with a controller P320. The lyophilisation of the PPy material was carried out with a lyophilisation device ALPHA 2-4 LSC from Christ.

2 Characterisation

2.1 SEM and EDX

Morphologies were observed by scanning electron microscopy (SEM) (Carl Zeiss CrossBeam 1540XB microscope with an accelerating voltage of maximum 3 kV). Energy-dispersive X-ray spectroscopy (EDX) measurements at this microscope were carried out with a INCA x-sight 7427 10 mm^2 from Oxford Instruments. Also, a Hitachi TM3000 Tabletop microscope (with an accelerating voltage of 15 kV and a Quantax EDX detector) was used for EDX measurements. The EDX measurements for the infiltration of $CuCl_2$ were carried out at a Zeiss Auriga Crossbeam Field Emission microscope (from Carl Zeiss with a Silicone Drift detector Oxford X-Max 20 mm^2 from Oxford Instruments). For SEM and EDX measurements, the specimen was sputtered with an ultrathin gold film as a conductive treatment (Edwards Scancoat Six Pirani 501 Sputter Coater from Edwards Laboratories).

2.2 TEM

The nanostructures were characterised by transmission electron microscopy (TEM) (Carl Zeiss Libra 120 microscope with an acceleration voltage of 120 kV).

2.3 Microtome

To embed the PPy material, a two-component epoxy resin glue (UHU plus endfest) was used. The embedded probes were cut with an ultramicrotome EMUC6 from Leica and a knife Cryotrim 45 from Diatome.

2.4 TGA

Thermal gravimetric analyses (TGA) were carried out with a Netzsch STA 449F3 Jupiter. The material was heated up from 30 °C to 90 °C with a heating rate of 10 K/min. The gas flow consisted of 254 mL/min oxygen and 250 mL/min nitrogen.

2.5 ATR-IR

Attenuated total reflection infrared spectroscopy (ATR-IR) was carried out with a Perkin Elmer Spectrum 100 FT-IR spectrometer.

2.6 BET

Brunauer-Emmett-Teller (BET) measurements were carried out with a Tristar from Micromeritics. The probes were pestled and degassed over night to remove adsorbed liquid or gas. The measurements were performed under nitrogen.

2.7 Preparation of electrodes

The working electrode was prepared by mixing electroactive material, carbon black and polyvinylidene fluoride (PVDF) with a weight ratio of 80:10:10 in N-methyl pyrrolidone (NMP) solvent to form a homogeneous slurry. Then, the prepared slurry was coated on to a graphite current collector (1 x 1 cm^2) and dried at 90 °C in air for 24 h to remove the solvent.

2.8 Electrochemical measurements

The electrochemical characteristics of the as-prepared electrodes were evaluated by using a conventional three electrode system in 1 M H_2SO_4 electrolyte solution. A platinum wire and Ag/AgCl were used as the counter and reference electrodes, respectively. The cyclic voltammetry, galvanostatic charge/discharge and impedance spectroscopy measurements were carried out using an Autolab PGSTAT302N electrochemical workstation. The specific capacitance (C) of the as-prepared electrode was calculated from the cyclic voltammetry (CV) curves according the following equation:

$$C = \frac{\int idV}{2vm\Delta V} \quad (1)$$

Where $\int idV$ is the integral area under the CV curve, v is the scan rate, m is the mass of the electroactive material (PPy) and $\triangle V$ is the potential window. Furthermore, the specific capacitance (C) was calculated from the galvanostatic charge/discharge curves according the following equation:

$$C = \frac{I\Delta t}{m\Delta V} \quad (2)$$

Whereby I is the discharge current and $\triangle t$ is the discharge time.

3 Figures

3.1 Contact angle (Figure S1)

Figure S1: Contact angles of the mixtures of 75 vol.-% Py and 25 vol.-% solvent (n-pentane, isopropanol, ethanol, methanol) plotted against the eluting power of the respective solvent from the elutropic series. The contact angle of pure Py is shown in the grey horizontal line. The contact angles have been measured in air on a calcite surface.

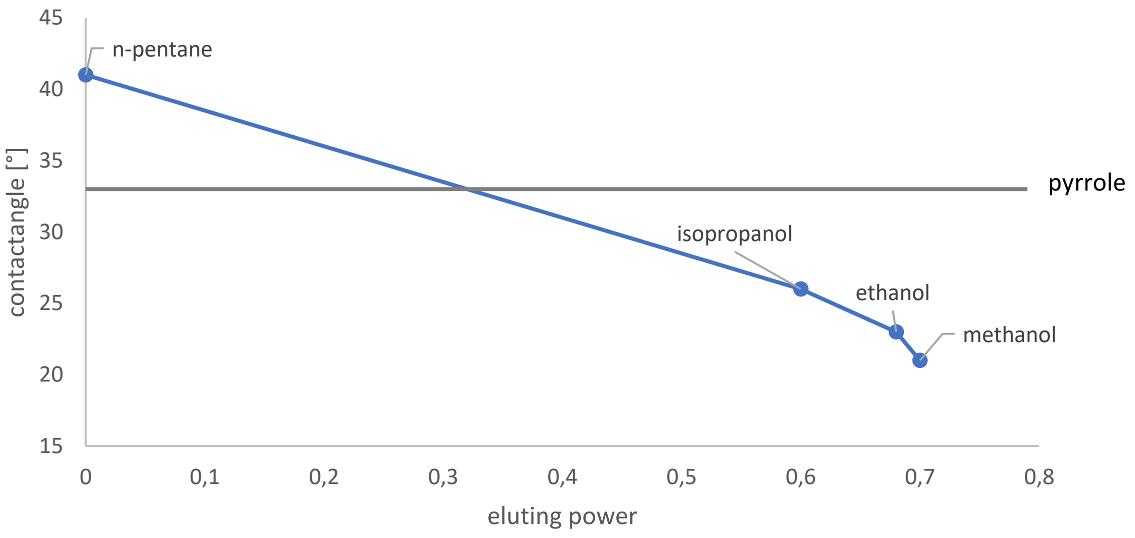

3.2 EDX analysis (Figure S2)

Figure S2: Graphical representation of the results of EDX measurements in at.-% for the sea urchin spine as template ($CaCO_3$), the composite material ($CaCO_3$, PPy) and the PPy after the dissolution of the template.

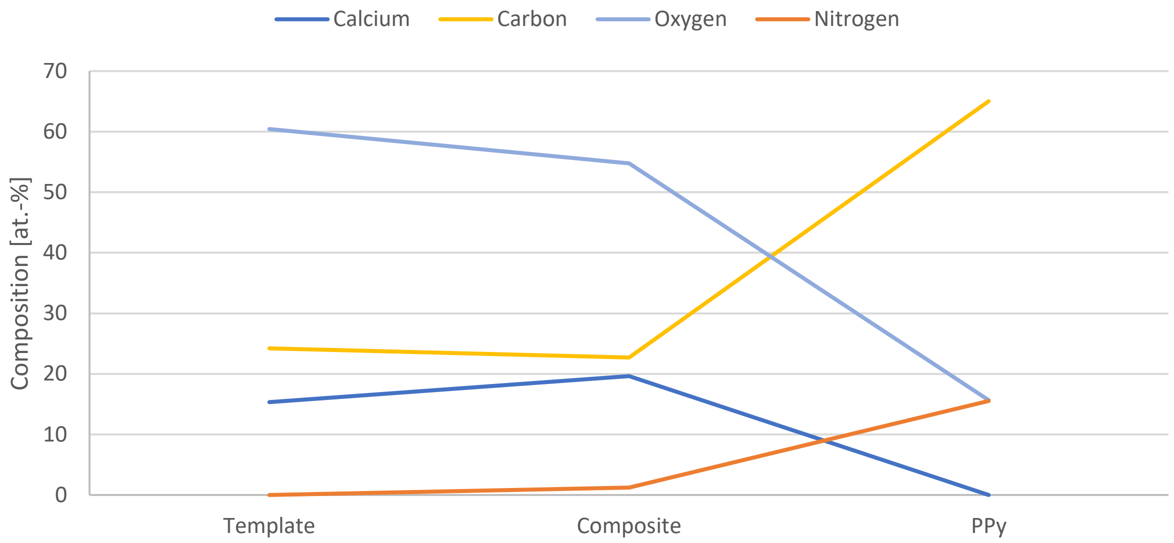

ATR-IR analysis (Figure S3, Figure S4)

Figure S3: ATR-IR spectra of the synthesised PPy (blue) and the template, the $CaCO_3$ of the sea urchin spine (black). The three characteristic peaks of $CaCO_3$ are non-existent in the spectra of PPy. A more detailed description of the peaks of PPy are given in Figure S4.

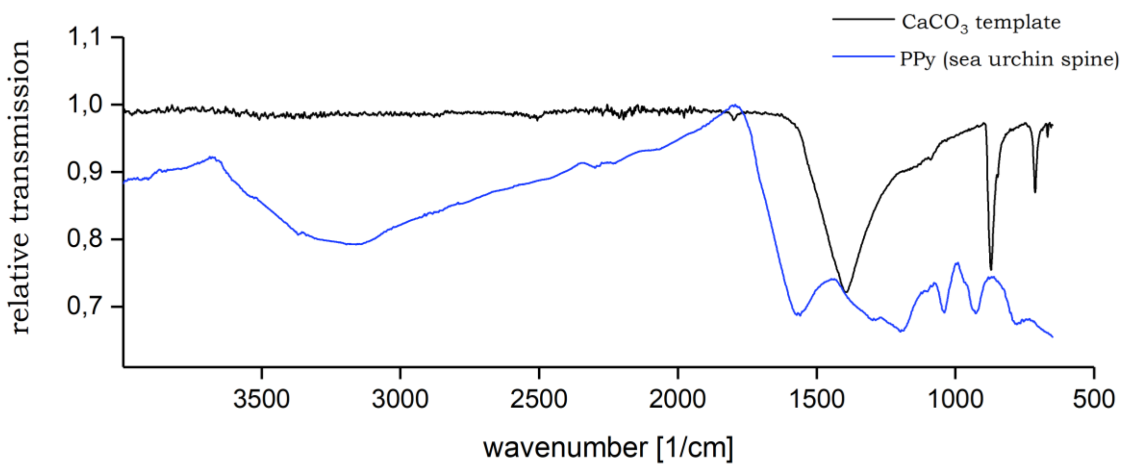

Figure S4: ATR-IR spectra of PPy synthesised in the sea urchin spine template (blue) and PPy synthesised under the same conditions in solution (black). For PPy the following peaks can be ascribed: 1560 cm^{-1} stretching vibration of C=C and C-C bonds (A), 1458 cm^{-1}stretching vibration of C=C and C-N bonds (B), 1299 cm^{-1} stretching vibration of C-C and C-N bonds (C), 1198 cm^{-1} stretching vibration of C-C bonds (D), 1040 cm^{-1} bending vibration of C-H bonds (E), 927 cm^{-1} stretching vibration of C=C and C-N bonds (F), 779 cm^{-1} bending vibration of C-H und N-H bonds (G), 3159 cm^{-1} stretching vibration of C-H and N-H bonds and hydroxide ions contained as counterion (H). (Assignment of the peaks according to Tian and Zerbi[1,2] as well as Imai et al.[3])

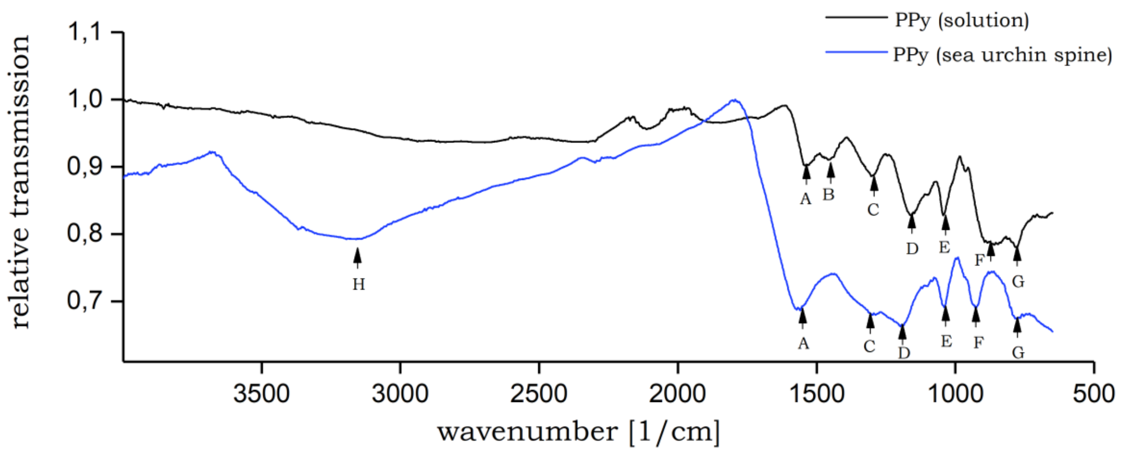

1. B. Tian and G. Zerbi, *The Journal of Chemical Physics*, 1990, **92**, 3886.
2. B. Tian and G. Zerbi, *Journal of Chemical Physics*, 1990, **92**, 3892.
3. M. Kijima et al., *Chemistry – A European Journal*, 2013, **19**, 2284.

3.3 TGA (Figure S5)

Figure S5: Results of TGA. The composite material consisting of $CaCO_3$ from the sea urchin spine template and PPy (blue) and the pure $CaCO_3$ from the sea urchin spine template (black). The enlarged part shows the additional decomposition step of the polymer. It was possible to infiltrate 1,38 wt.-% of PPy into the template.

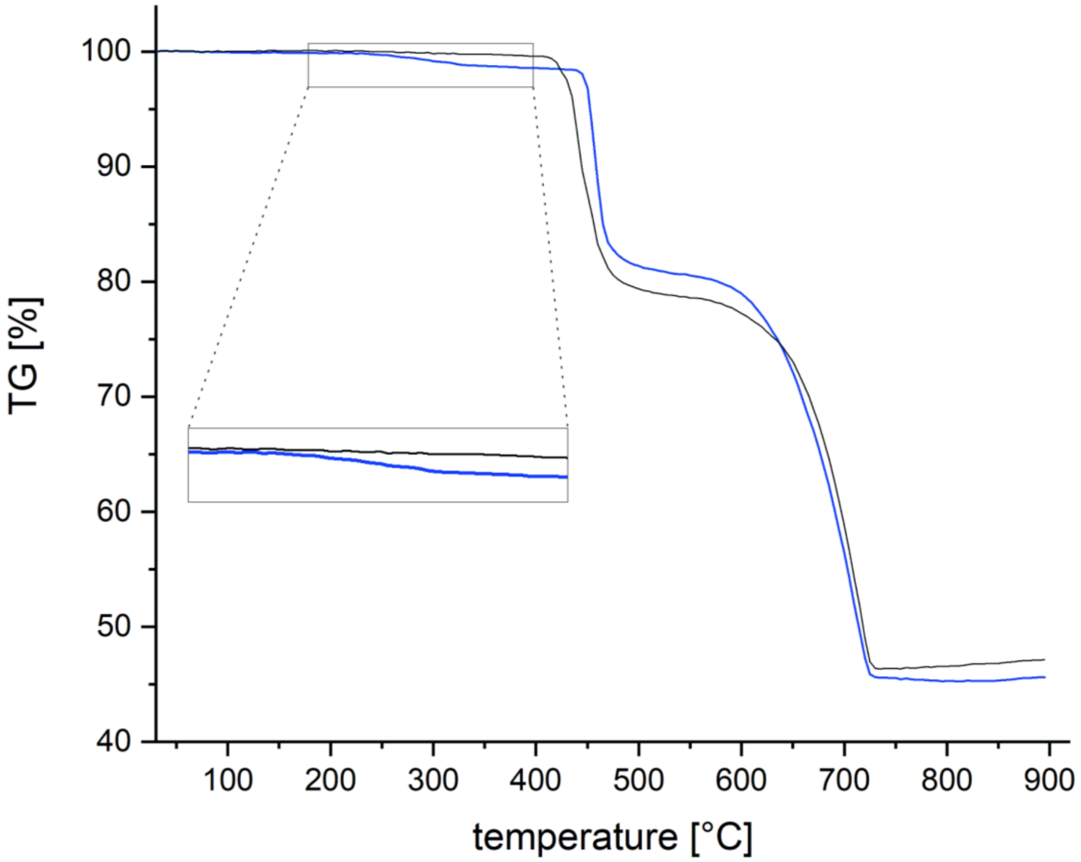

3.4 EDX mapping (Figure S6)

Figure S6: EDX measurement at the cross section of the sea urchin spine with infiltrated $CuCl_2$. a) The measurement was done in the middle part of the spine. The red box is shown enlarged in b). The measurement range is marked with the red box in b. c) Shows the mapping results of the EDX measurement for copper. The measurement confirms the infiltration of copper inside the sea urchin spine.

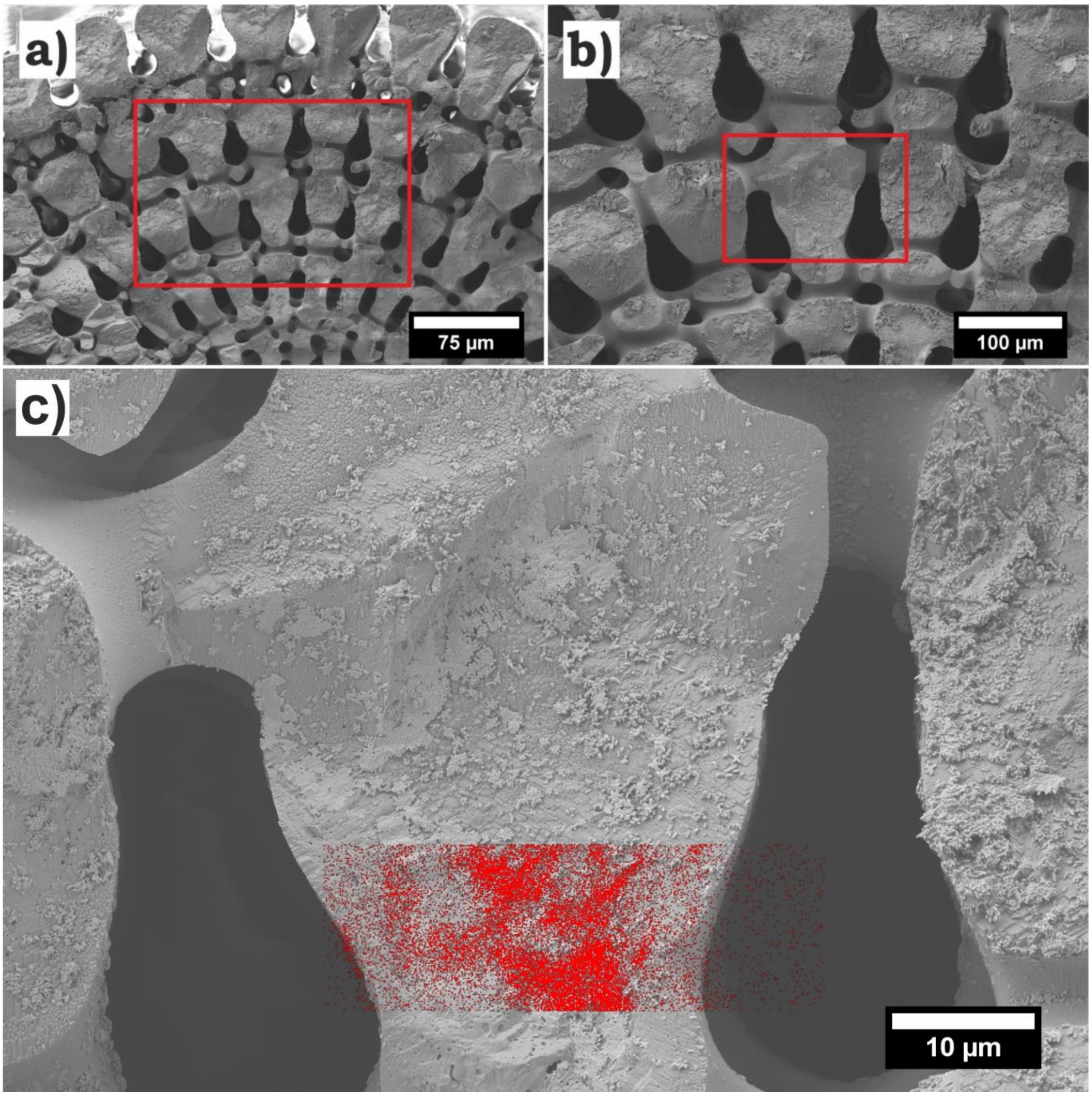

3.5 BET surface (Table S1)

Table S1: BET surface of PPy samples as result from physical adsorption measurements.

**Sample**	**BET surface** $[\frac{m^2}{g}]$
PPy synthesised in solution	13,3 ± 0,2
PPy synthesised after Imai et al.[1]	14,1 ± 0,4
PPy synthesised after the infiltration with MeOH	16,43 ± 0,07

1. Y. Oaki, M. Kijima and H. Imai, *Journal of the American Chemical Society*, 2011, **133**, 8594-8599.
